# Supplementary material for: The genome sequence of Triglochin palustris L., 1753 (Alismatales: Juncaginaceae)
Source: Wellcome Open Res. 2026 Jun 24;11:375. [Version 1] doi: 10.12688/wellcomeopenres.26845.1 (PMC13579031; doi:10.12688/wellcomeopenres.26845.1)
Supplement: Peer review history

# Reviewer report: Reviewer response for version 1

**DOI:** 10.21956/wellcomeopenres.29559.r166897

**Published:** 2026-09-15

**Authors:** Dmitry D. Sokoloff

**Affiliations:** 1 Tel Aviv University, Tel Aviv, Israel

This is a high quality and important study. *Triglochin palustre *is an interesting component of British flora that requires conservation.

 I have a few minor comments.

- **Background: **maybe add "in Britain and Ireland" about calcareous habitats, because it is not the case all throughout the range of the species. The phrase "In Britain and Ireland, the species has a circumpolar boreo-temperate distribution" looks strange, and here it is better to omit about Britain and Ireland.

I assume there are published cytological data on chromosome numbers in Triglochin palustre. I suggest citing them and comparing with the inferred number of pseudochromosomes. Are there published plastid genomes of this species? If yes, should they be compared to the new sequence? Should the two mitochondrial genomes be compared to what is known about mitogenome diversity in Alismatales? For example, Petersen, G., Cuenca, A., Zervas, A., Ross, G.T., Graham, S.W., Barrett, C.F., Davis, J.I. and Seberg, O., 2017. Mitochondrial genome evolution in Alismatales: Size reduction and extensive loss of ribosomal protein genes. *PLoS One*, *12*(5), p.e0177606.

Are sufficient details of methods and materials provided to allow replication by others?

Yes

Is the rationale for creating the dataset(s) clearly described?

Yes

Are the datasets clearly presented in a useable and accessible format?

Yes

Are the protocols appropriate and is the work technically sound?

Yes

Reviewer Expertise:

Monocot diversity and morphology

I confirm that I have read this submission and believe that I have an appropriate level of expertise to confirm that it is of an acceptable scientific standard.

## References

[1] : Mitochondrial genome evolution in Alismatales: Size reduction and extensive loss of ribosomal protein genes. *PLOS ONE* .2017;12(5) : 10.1371/journal.pone.0177606 10.1371/journal.pone.0177606 PMC543518528545148

# Reviewer report: Reviewer response for version 1

**DOI:** 10.21956/wellcomeopenres.29559.r166893

**Published:** 2026-09-08

**Authors:** Rogelio Sánchez Villegas

**Affiliations:** 1 Universidad Pablo de Olavide, Dos Hermanas Seville, Spain

The manuscript by Rushman et al. presents a high-quality genome assembly of *Triglochin palustris*. The manuscript is well written and well structured, and the figures and tables are appropriate and informative. Overall, the assembly pipeline follows approaches similar to those previously employed by the Wellcome Sanger Institute in other genome assembly projects, and the resulting assembly is of excellent quality. I only have two minor comments that I believe could further strengthen the manuscript:

 1. Hi-C data generated from a different individual: The authors state that the Hi-C data were generated from a different individual than the one used for the genome assembly. Could the authors provide a rationale for this choice? Using a different individual may introduce structural differences between genomes and could potentially explain the two small inversions reported on chromosomes 4 and 5. A brief discussion of this possibility would be valuable.

 2. BUSCO assessment:  For the BUSCO analysis, the authors used an OrthoDB v10 (odb10) reference dataset and recovered 86% complete BUSCOs, which is a reasonable level of completeness. However, it may be worth repeating the analysis using the more recent OrthoDB v12 (odb12) dataset.

Are sufficient details of methods and materials provided to allow replication by others?

Yes

Is the rationale for creating the dataset(s) clearly described?

Yes

Are the datasets clearly presented in a useable and accessible format?

Yes

Are the protocols appropriate and is the work technically sound?

Yes

Reviewer Expertise:

Plant Biology, Genetics, Chromosome Evolution

I confirm that I have read this submission and believe that I have an appropriate level of expertise to confirm that it is of an acceptable scientific standard.
